# Segurança Cardiovascular da Terapia de Reposição da Testosterona: Avaliação Crítica de um Ensaio Clínico Publicado Recentemente

**DOI:** 10.36660/abc.20230558

**Published:** 2024-03-21

**Authors:** Isabela Tramontini Muller, Sérgio Renato da Rosa Decker, Regis Goulart Rosa, Guilherme Rollin

**Affiliations:** 1 Hospital Moinhos de Vento Departamento de Medicina Interna Porto Alegre RS Brasil Hospital Moinhos de Vento – Departamento de Medicina Interna, Porto Alegre, RS –Brasil; 2 Hospital Moinhos de Vento Departamento de Endocrinologia Porto Alegre RS Brasil Hospital Moinhos de Vento – Departamento de Endocrinologia, Porto Alegre, RS – Brasil

**Keywords:** Doenças Cardiovasculares/prevenção e controle, Endocrinologia, Testosterona/tratamento farmacológico, Segurança do Paciente, Ensaios de Equivalência como Assunto

## Introdução

Na última edição de 16 de junho do *New England Journal of Medicin*, A. Michael Lincoff et al.,^1^ publicaram um estudo de não inferioridade, denominado "Segurança cardiovascular da terapia de reposição da testosterona" com o objetivo de determinar os resultados de segurança da terapia de reposição da testosterona em homens de meia-idade e idosos com hipogonadismo e doença cardiovascular pré-existente. Os critérios de inclusão para hipogonadismo foram sintomas e dois níveis séricos de testosterona em jejum inferiores a 300 ng/dL em amostras de sangue obtidas entre 5:00 h e 11:00 h. A intervenção foi a aplicação diária de gel transdérmico de testosterona a 1,62% com ajustes para manter os níveis de testosterona entre 350 e 750 ng/dL ou responder a um hematócrito superior a 54%.

As características de linha de base incluíam homens, com idade média de 63 anos, dos quais 80% eram brancos, com índice de massa corporal médio de 35, e entre os quais 55% tinham doença cardiovascular pré-existente. O nível mediano de testosterona foi de 227 ng/dL em ambos os grupos, e o aumento médio em relação ao valor de linha de base foi de 148 ng/dL no grupo de testosterona, em comparação com um aumento médio de 14 ng/dL no grupo de placebo. O estudo concluiu a não inferioridade para eventos cardíacos adversos maiores (MACE) comparando a reposição de testosterona com placebo, e uma margem de não inferioridade pré-especificada de 1,5. Os resultados finais mostraram uma razão de risco para o desfecho primário de 0,96 (intervalo de confiança de 95%, 0,78-1,17; p-valor para não inferioridade <0,001).^1^ No entanto, temos preocupações em relação a essa alegação de não inferioridade, principalmente devido a dois pontos: a margem de não inferioridade e a escolha de um MACE de três pontos em vez de um MACE de cinco pontos.^2,3^

### Descrição

O MACE como desfecho composto melhora o poder de detectar diferenças em ensaios clínicos e tem sido defendido pela Federal Drug Administration para ensaios envolvendo medicamentos para diabetes.^4^ Contudo, para obter maior sensibilidade, devem ser considerados os desfechos que compreendem alterações nos MACE, levando em consideração o efeito esperado por meio de um MACE de cinco pontos (morte, infarto do miocárdio, acidente vascular cerebral, revascularização da lesão-alvo ou eventos tromboembólicos e internações por insuficiência cardíaca).

As evidências sugerem que, para determinar com segurança uma margem de não inferioridade, ela deve ser estabelecida com base em resultados anteriores sugeridos por estudos de superioridade sobre o mesmo tema.^2,3^ O que foi estabelecido é que as margens de não inferioridade devem abranger eventos considerados importantes em estudos de superioridade.^2,3^ Em uma coorte retrospectiva publicada no JAMA em 2013, que avaliou um MACE de três pontos comparando testosterona com placebo, observou-se que o grupo testosterona teve um risco aumentado de mortalidade global, infarto do miocárdio e acidente vascular cerebral (RC 1,29; IC 95% 1,05-1,58; p = 0,02).^5,6^ No entanto, as evidências de ensaios clínicos randomizados que avaliam a reposição de testosterona são escassas, e, portanto, os autores utilizaram suposições para a margem de não inferioridade, escolhendo uma razão de risco de 1,5 como margem. Nesse caso, num exemplo fictício, se a população estudada tiver uma taxa de incidência de MACE de 10% a margem de 1,5, uma taxa de até 15% (10% + 5%) seria considerada aceitável como "segura" em relação à não inferioridade do novo medicamento; esse valor foi superior ao normalmente utilizado em ensaios de não inferioridade em cardiologia, que geralmente apresentam uma média de 1,3 (IC 95% 1,2-1,4) em termos relativos.^2,3^

Na publicação, as variáveis consideradas para MACE foram as três seguintes: primeira ocorrência de qualquer componente de um composto de morte cardiovascular, infarto do miocárdio não fatal ou acidente vascular cerebral não fatal. Uma revisão publicada descobriu que apenas alguns estudos utilizam o MACE tradicional de três pontos.^5^ Como o resultado composto ocorre com mais frequência do que os seus componentes individuais, um resultado composto pode reduzir o número de participantes necessários num estudo.^7^ Especialmente num ensaio de não inferioridade, o poder de detectar resultados é crucial. No caso do ensaio sobre testosterona, as hospitalizações por insuficiência cardíaca, revascularização urgente e eventos tromboembólicos são importantes e ofereceriam uma visão mais abrangente dos resultados cardiovasculares, incluindo eventos clinicamente relevantes. Na realidade, a incidência de embolia pulmonar foi mais alta com a testosterona do que com placebo (RR 1,46 (0,92 - 2,32).^1^

Em 2010, o estudo TOM, que randomizou homens com mais de 65 anos com limitações de mobilidade e níveis séricos totais de testosterona entre 100 e 350 ng/dL ou níveis de testosterona livre inferiores a 50 pg/mL para receber placebo ou gel de testosterona com o objetivo de demonstrar ganho de massa muscular no grupo testosterona, teve que ser interrompido prematuramente devido a uma maior incidência de eventos adversos cardiovasculares no grupo da intervenção.^8^ Este estudo utilizou um MACE mais abrangente, incluindo infarto do miocárdio, síndrome coronária aguda, arritmias, insuficiência cardíaca congestiva e morte súbita. Na avaliação de cada evento adverso, o estudo não teve poder suficiente para mostrar um risco aumentado para o grupo da testosterona. Entretanto, ao considerar o resultado cardíaco composto, houve 22% versus 5% mais eventos no grupo da testosterona (p<0,001).^8^ Isso destaca a importância de abordar as principais configurações de resultados dos estudos, principalmente no que diz respeito a questões de segurança.

Finalmente, ao incorporar esses resultados no MACE composto e usar uma margem de não inferioridade mais conservadora (RR 1,3), provavelmente a terapia de reposição hormonal com testosterona seria inferior ao placebo e seu uso deve ser considerado com cautela.
