# Supplementary material for: Segurança Cardiovascular da Terapia de Reposição da Testosterona: Avaliação Crítica de um Ensaio Clínico Publicado Recentemente
Source: Arq Bras Cardiol. 2024 Mar 21;121(3):e20230558. [Article in Portuguese] doi: 10.36660/abc.20230558 (PMC11081064; doi:10.36660/abc.20230558)
Supplement: Translation

# Translation: Cardiovascular Safety of Testosterone-Replacement Therapy: Critical Appraisal of a Currently Published Clinical Trial

**DOI:** 10.36660/abc.20230558i

**Authors:** Isabela Tramontini Muller, Sérgio Renato da Rosa Decker, Regis Goulart Rosa, Guilherme Rollin

**Affiliations:** 1 Porto Alegre RS Brazil Hospital Moinhos de Vento – Departamento de Medicina Interna, Porto Alegre, RS – Brazil; 2 Porto Alegre RS Brazil Hospital Moinhos de Vento – Departamento de Endocrinologia, Porto Alegre, RS – Brazil

**Keywords:** Testosterone, Cardiovascular, Outcomes, Non-inferiority, Safety

## Introduction

In the last edition on June 16 of the New England Journal of Medicine, A. Michael Lincoff et al.^1^ published a non-inferiority trial, entitled "Cardiovascular Safety of Testosterone-Replacement Therapy", aiming to determine the safety outcomes of testosterone replacement therapy in middle-aged and older men with hypogonadism and pre-existing cardiovascular disease. The inclusion criteria for hypogonadism were symptoms and two fasting serum testosterone levels of less than 300 ng/dL in blood samples obtained between 5:00 a.m. and 11:00 a.m. The intervention was daily transdermal 1.62% testosterone gel with adjustments to maintain testosterone levels between 350 and 750 ng/dL or to respond to a hematocrit greater than 54%.

The baseline characteristics were men, with a mean age of 63 years, 80% were white, with a mean body-mass index of 35, and 55% had preexisting cardiovascular disease. The median testosterone level was 227 ng/dL in both groups, and the median increase from baseline was 148 ng/dL in the testosterone group, as compared to a median increase of 14 ng/dL in the placebo group. The study concluded non-inferiority for major adverse cardiac events (MACE) by comparing testosterone replacement to placebo, and a prespecified non-inferiority margin of 1.5. The final results showed a hazard ratio for the primary end-point of 0.96 (95% confidence interval, 0.78-1.17; p-value for non-inferiority <0.001).^1^ However, we have concerns regarding this claim of non-inferiority mainly because of two points: the margin of non-inferiority and the choice of a three-point MACE over a five-point MACE.^2,3^

### Descrição

MACE as a composite endpoint improves the power to detect differences in clinical trials and has been advocated by the Federal Drug Administration for trials involving diabetes medications.^4^ However, to achieve enhanced sensitivity, the outcomes comprising changes in MACE should be considered, taking into account the expected effect through a five-point MACE (death, myocardial infarction, stroke, target lesion revascularization or thromboembolic events, and heart failure hospitalizations).

Evidence suggests that to safely determine a margin of non-inferiority, it should be established based on previous results suggested by superiority studies on the same topic.^2,3^ What has been established is that non-inferiority margins should encompass events considered important in superiority studies.^2,3^ In a retrospective cohort published in JAMA in 2013, which evaluated a three-point MACE comparing testosterone with placebo, it was observed that the testosterone group had an increased risk of all-cause mortality, myocardial infarction, and stroke (OR 1.29; 95% CI 1.05-1.58; p = 0.02).^5,6^ However, the evidence from randomized clinical trials assessing testosterone replacement is scarce; thus, the authors used assumptions for non-inferiority margin, choosing a hazard ratio of 1.5 as the margin. In this case, in a fictitious example, if the studied population has an incidence rate of MACE of 10% the margin of 1.5, a rate of up to 15% (10% + 5%) would be considered acceptable as "safe" concerning the non-inferiority of the new medication; this was higher than the one typically used in non-inferiority trials in cardiology, which generally present an average of 1.3 (95% CI 1.2-1.4) in relative terms.^2,3^

In the publication, the variables considered for MACE were the following three: the first occurrence of any component of a composite of cardiovascular death, non-fatal myocardial infarction, or non-fatal stroke. A published review found that only a few studies use the traditional three-point MACE.^5^ As the composite outcome occurs more frequently than its individual components, a composite can reduce the number of participants needed in a study.^7^ Especially in a non-inferiority trial, the power to detect outcomes is crucial. In the case of the testosterone trial, hospitalizations for heart failure, urgent revascularization, and thromboembolic events are important and would provide a more comprehensive view of cardiovascular outcomes, including clinically relevant events. Indeed, the incidence of pulmonary embolism was higher with testosterone than with the placebo (HR 1.46 (0.92 - 2.32).^1^

In 2010, the TOM study, which randomized men over 65 years with mobility limitations and total serum testosterone levels between 100 and 350 ng/dL or free testosterone levels less than 50 pg/mL to receive either a placebo or testosterone gel with the aim of demonstrating muscle mass gain in the testosterone group, had to be prematurely terminated due to a higher incidence of cardiovascular adverse events in the intervention group.^8^ This study had used a more comprehensive MACE, including myocardial infarction, acute coronary syndrome, arrhythmias, congestive heart failure, and sudden death. When evaluating each adverse event, the study was underpowered to show an increased risk for the testosterone group. However, when considering the composite cardiac outcome, there were 22% versus 5% more events in the testosterone group (p<0.001).^8^ This underscores the significance of addressing studies’ core outcome settings, mainly regarding safety concerns.

Finally, by incorporating these outcomes into the composite MACE and using a more conservative non-inferiority margin (OR 1.3), testosterone hormone replacement therapy would most likely be inferior to placebo and its use should be considered with caution.
